# Lagos state ambulance service: a performance evaluation

**DOI:** 10.1007/s00068-020-01319-y

**Published:** 2020-03-10

**Authors:** Chinmayee Venkatraman, Aina Olufemi Odusola, Chenchita Malolan, Olusegun Kola-Korolo, Oluwole Olaomi, Jide Idris, Fiemu E. Nwariaku

**Affiliations:** 1 Office of Global Health, Department of Surgery, University of Texas Southwestern Medical Center, 5323 Harry Hines Boulevard, Dallas, TX 75390 United States of America; 2 Department of Community Health & Primary Health Care, Lagos State University Teaching Hospital, 1—5, Oba Akinjobi Road, Ikeja, Lagos, Nigeria; 3 Lagos State Ministry of Health, Block 4, The Lagos State Government Secretariat Complex, Alausa, Ikeja, Lagos, Nigeria; 4 Department of Surgery, National Trauma Centre, National Hospital Abuja, Plot 321, Central Business District, FCT, Abuja, Nigeria

**Keywords:** Road traffic injuries, Mortality, Nigeria, Emergency medical services, Prehospital care

## Abstract

**Objectives:**

The mortality rate from road traffic accidents (RTAs) in Nigeria is almost double that of the USA. In Nigeria, the first emergency medical services (EMS) system was established in March 2001, The Lagos State Ambulance Service (LASAMBUS). The objectives of this study are to (1) determine the burden of RTAs in Lagos, (2) assess RTA call outcomes, and (3) analyze LASAMBUS’s response time and causes for delay.

**Methodology:**

We reviewed completed LASAMBUS intervention forms spanning December 2017 to May 2018. We categorized the call outcomes into five groups: I. Addressed Crash, II. No Crash (False Call), III. Crash Already Addressed, IV. Did Not Respond, and V. Other. We further explored associations between the (1) causes for delay and outcomes and (2) response times and the outcomes.

**Results:**

Overall, we analyzed 1352 intervention forms. We found that LASAMBUS did not address 53% of the RTA calls that they received. Of this, Outcome II. No Crash (False Call) accounted for 26% and Outcome III. Crash Already Addressed accounted for 22%. Self-reported causes for delay were recorded in 180 forms, representing 13.7% of the RTA burden. Traffic congestion accounted for 60% of this distribution.

**Conclusion:**

LASAMBUS response rates are significantly lower than response rates in high-income countries such as the USA and lead to increased RTA mortality rates. Eliminating causes for delay will improve both LASAMBUS effectiveness and RTA victims’ health outcomes. Changing the public perception of LASAMBUS and standardizing LASAMBUS’ contact information will aid this as well.

**Electronic supplementary material:**

The online version of this article (10.1007/s00068-020-01319-y) contains supplementary material, which is available to authorized users.

## Introduction

The global prevalence of road traffic accidents (RTAs) and road traffic injuries (RTIs) is steadily increasing. According to the World Health Organization, RTAs killed 1.35 million people in 2018 and injured an additional 50 million [1]. RTIs are now the leading cause of death among children and young adults aged 5–29 years, overtaking HIV/AIDS, diarrheal diseases, and tuberculosis [2]. This burden is disproportionately higher in low- and middle-income countries (LMICs), with 93% of road traffic fatalities occurring in these settings [1, 3]. Globally, road traffic fatality rates are the highest in the African continent at 26.6 deaths per 100,000 [4]. Nigeria has an annual mortality rate of 20.6 deaths per 100,000 people due to RTAs, in comparison to the USA at 10.8 deaths per 100,000 people and the UK at 2.9 deaths per 100,000 people [5].

Lagos is the most densely populated state in Nigeria (6710 population per km^2^), which is more than three times the population density of New Jersey (1947 population per km^2^), the most densely populated state in the USA [6, 7]. Lagos is divided into 20 local government areas (LGAs) and has an intricate system of road networks managed by various levels of government. Trunk A roads are maintained by the federal government, Trunk B by the state government, and local roads by the local government with aid from the state government [8]. Additionally, there are several major intra- and interstate expressways throughout Lagos. Coordinating infrastructure management within these levels of government is difficult and often leads to poor road conditions [8]. One major concern is the presence of numerous potholes across all types of roads, sometimes large enough to cover more than half the width of the road [9]. In fact, in 2012, 81% of the roads examined in Lagos had more than 100 potholes, resulting in unsafe road conditions [9]. The Lagos State Public Works Corporation (PWC) is the government entity “responsible for routine repair and rehabilitation of road across the state, such that they remain motorable all year round” [10]. It coordinates road reconstruction across the state and works with local governments to identify specific issues. One major issue that it encounters is the weather in Lagos, specifically the rainy season. The Lagos climate is generally high in humidity with high temperatures, with the exception of a rainy season from June to October [11]. Not only does this primarily affect the repairs of potholes in the roads, but it also creates drainage issues that further delay these repairs, affecting motor vehicle and pedestrian travel, RTA rates, RTA response times, and prehospital care delivery [12].

Emergency medical services (EMS) systems are an essential part of prehospital management of RTIs. Increased EMS response times have been proven to be associated with higher mortality rates in rural communities [13]. The median urban response time in Africa is 15 min (6–120 min), which is more than double the median urban response time in the USA [14, 15]. Currently in Africa, there are 25 EMS systems in 16 countries, representing merely 30% of the continent [15]. West Africa is especially underrepresented with EMS systems only present in Ghana and Nigeria [15]. Oftentimes, the lack of a national prehospital trauma care system results in EMS systems established by state governments or private corporations. This, in turn, leads to a lack of standardized prehospital care delivery within the country [16]. In fact, a majority of these systems only provide ambulance transport services as opposed to both transport and paramedic services [16]. For example, an EMS system in Imo State is staffed entirely by volunteers who are not trained to provide prehospital care [16]. Contrastingly, in Lagos State, the state government has invested in the Lagos State Ambulance Service (LASAMBUS), which is better equipped to attend to emergency situations [16].

LASAMBUS was established in Lagos in March 2001 as the first EMS system in Nigeria [17]. There are three main EMS systems in Lagos: LASAMBUS, Lagos State Emergency Management Agency (LASEMA), and Lagos Response Unit (LRU). LASAMBUS uses standard ambulances and there are currently 25 ambulance stations in the state. When someone calls for an ambulance in the event of an RTA or other accidents, the call is received by a call center in Lagos, which dispatches the ambulance closest to the crash site. Concurrently, LASAMBUS completes an intervention form detailing the response from when the call was received to when it was concluded. LASAMBUS then transports the RTA victims to a nearby hospital. Lagos has two main trauma care centers, The Lagos State Accident and Emergency Centre and the Burns and Trauma Unit at Gbagada General Hospital. LASAMBUS receives 11,126 calls annually, ranging from trauma cases and general medical cases to hospital transfers. In 2012, an assessment of LASAMBUS found that RTAs accounted for the largest proportion of calls received [17]. Additionally, traffic congestion and community disturbance were listed as causes for delay that LASAMBUS encountered [17]. The objectives of this study were to:

1. Determine the burden of RTAs in Lagos State.
2. Assess the RTA call outcomes.
3. Analyze LASAMBUS’s response time and causes for delay.

## Methods

This is a retrospective, cross-sectional study. We received completed LASAMBUS intervention forms that were classified as RTA calls from December 2017 to May 2018 from the Lagos State Ministry of Health. We omitted 10.1% of the forms based on our exclusion criteria, which included any LASAMBUS call that were misclassified as an RTA, any that were not in the study time frame, or any that were intervention forms in which the first and second pages of the form did not pertain to the same call scenario (missing pages, blank pages, etc.). After applying our exclusion criteria, we reviewed 1352 intervention forms.

Electronic supplementary material: Appendix A is a blank version of the intervention form. We focused our analyses on the following sections:

- Date of Call
- Timing of Call
- Demographics of the Victim
- Distribution of Cases
- Intervention and Monitoring
- Trauma Prompts
- Causes for Delayed Response
- Triage Revised Trauma Score
- Remarks of the LASAMBUS Crew

To determine the outcomes of the calls received, we reviewed the “Remarks” section of the forms, which was written as a narrative. We categorized the responses into five outcomes:

I. Addressed Crash.
II. No Crash (False Call).
III. Crash Already Addressed.
IV. Did Not Respond.
V. Other.

We further categorized certain outcomes based on common findings. The forms were hand-written and while we acknowledge the possibility that forms could have been illegible, we did not encounter any illegible forms.

An electronic version of the LASAMBUS form was created to manage study data using the REDCap electronic data capture tool [18]. To this form, we added the Outcomes section, a second Trauma Prompts section, and a second Causes for Delay section. The latter two sections were created to account for those forms that had a specific trauma prompt or cause for delay mentioned in the “Remarks”, but were not appropriately marked in the respective sections of the intervention forms. Since we were able to accurately identify these, we combined the data from the form along with what should have been marked initially for both the Trauma Prompts section and the Causes for Delay section for all subsequent analyses. Response Time was defined as the difference between when the call was received and when LASAMBUS arrived at the RTA site. We encountered some missingness in the data with regard to our response time analyses. We employed a pairwise deletion analysis technique to account for those observations that only had a call received time or a time when LASAMBUS arrived at the RTA site, for which we could not calculate a response time. We were able to successfully calculate a response time in 82.6% of cases. Stata 15 was used to conduct descriptive statistical analysis and logistic regression analysis where *α* = 0.05. Bivariate analyses were conducted to assess the association between Causes for Delay and each Outcome. Multivariate regression analyses evaluated the relationship between significant Causes for Delay and all Outcomes, and the relationship between Response Time and all Outcomes.

The University of Texas Southwestern Medical Center’s Institutional Review Board approved this study as non-regulated research, citing the U.S. Department of Health & Human Services’ regulation 45 CFR 46.102. The NIH Partnerships to Develop Injury Research Capacity in Sub-Saharan Africa grant (5D43TW010463-03) supported this research.

## Results

**Fig. 1:**
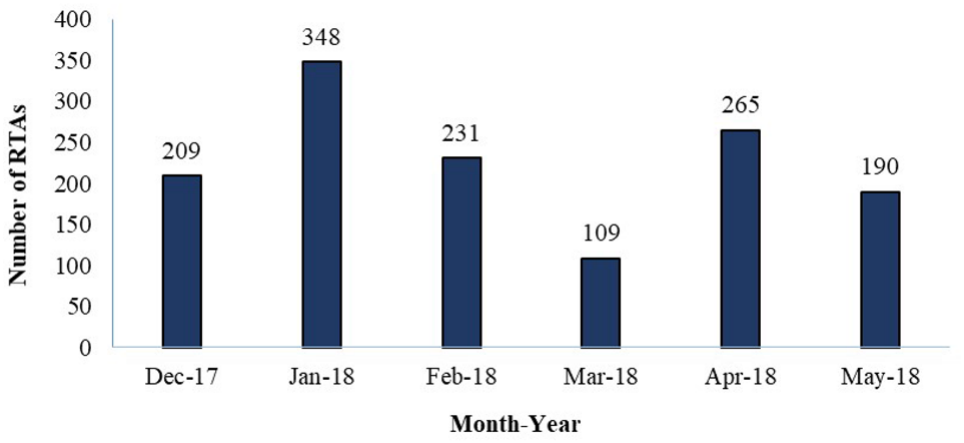
Monthly distribution of RTA calls in Lagos State received by LASAMBUS, December 2017–May 2018

**Fig. 2:**
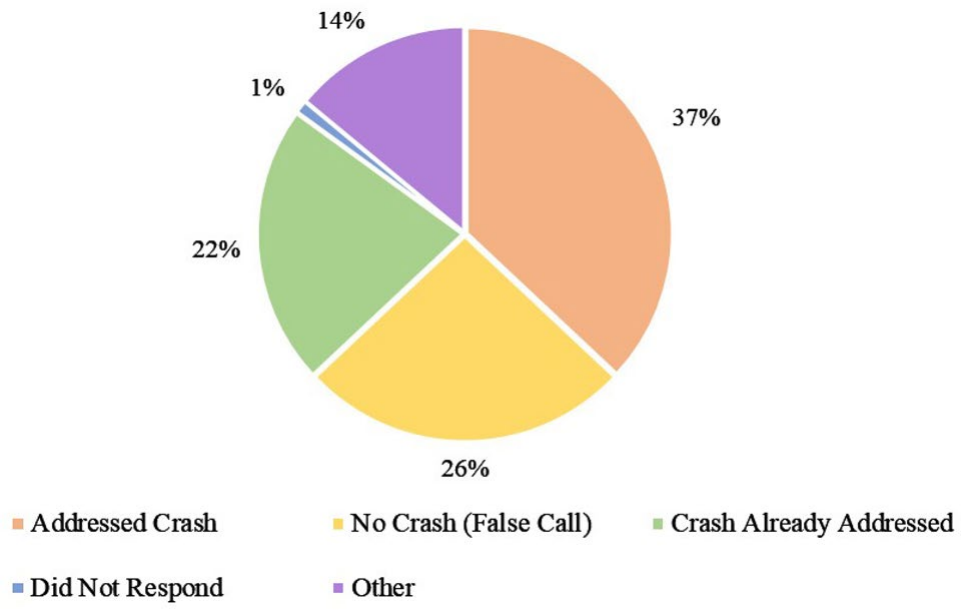
Distribution of outcomes of RTA calls received by LASAMBUS, December 2017–May 2018

**Fig. 3:**
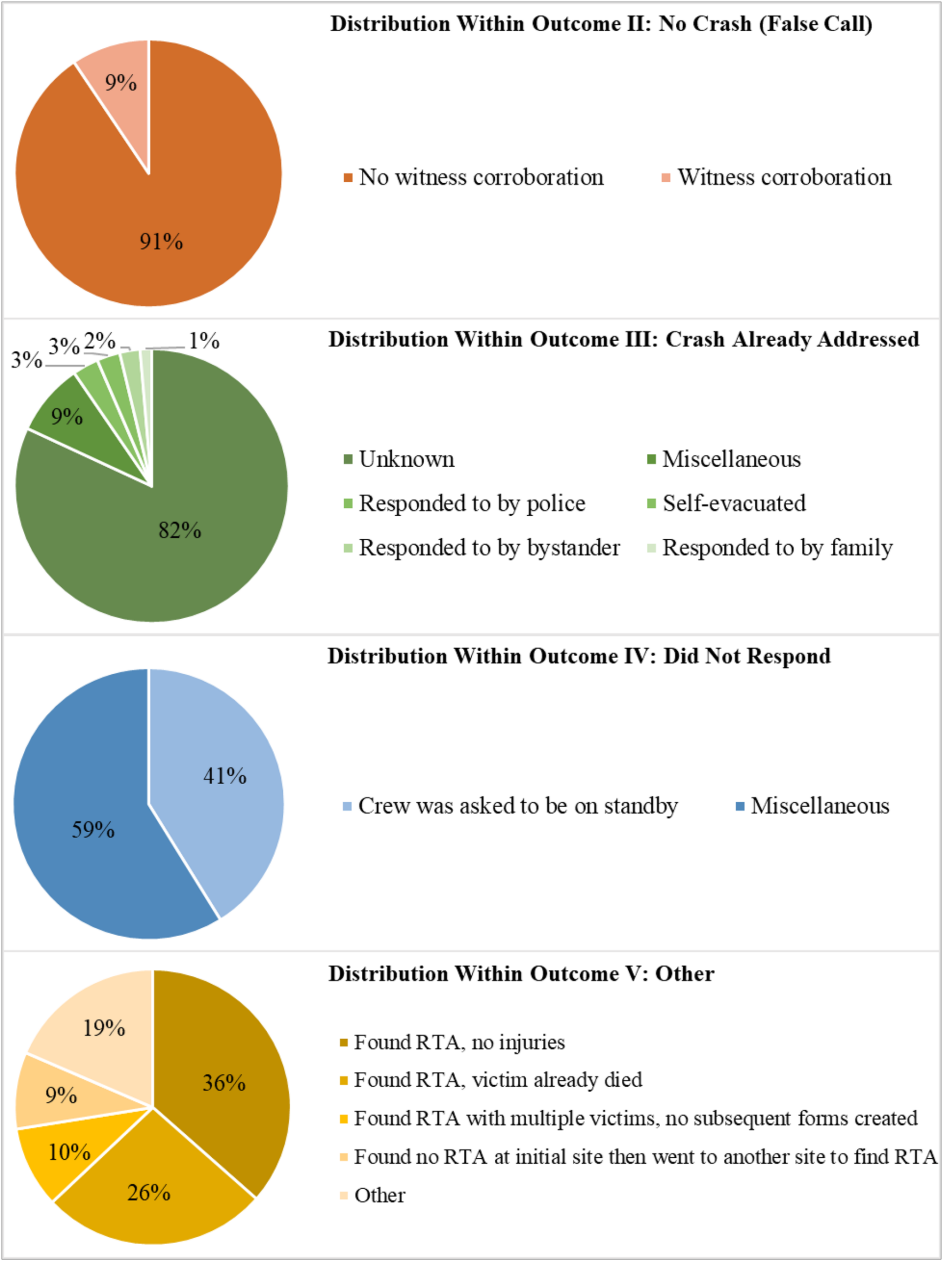
Distribution of responses within outcomes of RTA calls received by LASAMBUS, December 2017–May 2018

LASAMBUS received 1352 RTA calls between December 2017 and May 2018. Figure 1 shows the monthly distribution of calls received during the study period, with an average of 226 calls per month. Table 1 displays the descriptive characteristics of the dataset. The median age of the RTA victims was 34.0 years (SD 12.0) and the majority (73%) were male. The average response time of each LASAMBUS call was 17.0 min (7–60 min). We were able to ascertain the outcome of every call, as there were no illegible forms. LASAMBUS only addressed 37.1% of the calls that they received (Outcome I). Outcome II: No Crash (False Call) and Outcome III: Crash Already Addressed represented almost 50% of the call outcomes (Fig. 2). We found common responses in these categories that we further coded into subcategories (Fig. 3). Outcome II: No Crash (False Call) defined calls in which no crash was sighted, with or without witness corroboration. Only 9.4% of the false calls had witness corroboration. Within Outcome III: Crash Already Addressed, the most common sub-category was “Unknown” (81.9%), in which the only description LASAMBUS gave was “crash was already addressed”. This was followed by “Responded to by Police” (3.1%) and “Self-Evacuated” (2.7%). “Miscellaneous” responses for Outcome III included “attended to by LASEMA” and “attended to by LRU”. Within Outcome IV: Did Not Respond, “crew was asked to be on standby” represented 41.4% of the calls. “Miscellaneous” responses included “no fuel” and “no ambulance available”. Within Outcome V: Other, “found RTA, no injuries” (36.5%) and “found RTA, victim already died” (26.5%) accounted for over half of the responses.

**Table 1:** Descriptive characteristics of RTAs attended to by LASAMBUS, December 2017–May 2018

	Total sample (*n* = 1352) n (%)
**Age (years)** **(*n*** **= 437)**	34.0 (3.0–85.0)
**Gender** **(*n*** **= 466)**	
Male	340 (73.0)
Female	126 (27.0)
**Response time**	
“Call received” to “arrived at scene” (minutes)	17.0 (7.0–60.0)
**Distribution of outcomes**	
Outcome I: addressed crash	502 (37.1)
Outcome II: no crash (false call)	351 (26.0)
Outcome III: crash already addressed	293 (21.7)
Outcome IV: did not respond	17 (1.3)
Outcome V: other	189 (14.0)

Table 2 shows the distribution of self-reported causes for delay and the Fisher’s exact analyses for Causes for Delay and all Outcomes. Causes for delay were reported in 180 forms, representing 13.7% of the RTA burden. Traffic congestion accounted for 60% of the distribution, followed by poor description (17.8%) and proximity (7.2%). Furthermore, traffic congestion (*p* = 0.001), poor access (*p* < 0.0001), and community disturbance (*p* = 0.016) all had a significant association with the Outcomes. Table 3 shows the multivariate regression analyses for (1) Traffic Congestion and all Outcomes and (2) Poor Access and all Outcomes. We did not conduct regression analyses for causes for delay if the association between the cause for delay and all outcomes was not significant in the Fisher’s exact analyses or if there were less than five observations of a particular cause for delay, which is not compatible with regression analysis. For Traffic Congestion, we found a significant association with Outcome III (*p* = 0.011) and Outcome IV (*p* = 0.026). For Poor Access, we only found a significant association with Outcome IV (*p* = 0.001). Table 4 shows the multivariate regression analyses for Response Time and the Outcomes, which did not yield any significant associations.

**Table 2:** Evaluating the bivariate relationship between each cause for delay and all outcomes (*n* = 180)

Cause for delay	Distribution (%)	Fisher’s exact test
Traffic congestion	108 (60.00)	0.001*
Poor description	32 (17.78)	0.190
Proximity	13 (7.22)	0.226
Poor access	9 (5.00)	0.000*
Faulty ambulance	9 (5.00)	0.100
Community disturbance	4 (2.22)	0.016*
Other	4 (2.22)	0.105
Weather	1 (0.56)	0.629

^*^p value < 0.05

**Table 3:** Evaluating multivariate relationship between significant causes for delay from Table 2 and all outcomes

Outcomes	Causes for delay, *p* value	
	Traffic congestion	Poor access
Outcome I: addressed crash	Ref	
Outcome II: no crash (false call)	0.060	0.784
Outcome III: crash aready addressed	0.011*	0.899
Outcome IV: did not respond	0.026*	0.052
Outcome V: other	0.001*	0.816

^*^p value < 0.05

**Table 4:** Evaluating multivariate relationship between response time and all outcomes

Outcomes	Response time, *p* value
Outcome I: addressed crash	Ref
Outcome II: no crash (false call)	0.0925
Outcome III: crash already addressed	0.600
Outcome IV: did not respond	0.380
Outcome V: other	0.185

^*^p value < 0.05

## Discussion

Through this study, we identified three key findings:

1. There was variance in the monthly distribution of RTAs to which LASAMBUS attended.
2. LASAMBUS did not address more than 50% of the RTA calls they received.
3. There were significant associations between specific Causes for Delay and Outcomes—a. Poor Access and Outcome IV: Did Not Respond, b. Traffic Congestion and Outcome III: Crash Already Addressed and c. Traffic Congestion and Outcome IV: Did Not Respond.

Pre-hospital care management is integral to improving patient outcomes, particularly victims of RTAs. Previous studies have shown that mortality rates could be up to 5.5 times higher in RTA victims without pre-hospital care [19]. By characterizing the cases attended to by LASAMBUS, identifying the outcomes of the calls, and recognizing the scenarios leading to these outcomes, we are better informed about the pre-hospital care management that LASAMBUS provides.

A number of our findings were consistent with existing literature. Our victim population demographics resembled those reported by the World Health Organization, other studies in Nigeria, and in other LMICs such as Iran [3, 20, 21]. With regard to the second finding, our study showed that LASAMBUS attended to less than half of the RTA calls that it received. Similarly, a 2017 single-institution study revealed that, of all the RTAs that came into the emergency department of a tertiary health facility, the Lagos State University Teaching Hospital (LASUTH), less than 3% were brought in by LASAMBUS [22].

The outcomes of the calls that LASAMBUS received provide insights into the burden of RTAs, notably the prevalence of false calls and the RTAs that had been addressed prior to LASAMBUS’ intervention. There is a dearth of literature concerning false calls in LMICs; however, they have been identified as a challenge in an EMS study conducted in Ghana [23]. With regard to RTAs that were already addressed prior to LASAMBUS’ arrival, findings in other LMICs reinforce the idea that EMS services do not account for many of the cases brought into the hospital [24]. It is important to acknowledge that not all RTAs resulted in injury and that there were also records in which the victims had already died prior to LASAMBUS’ intervention.

Ambiguity around how to contact EMS systems, like LASAMBUS, can exacerbate the problem. A survey done in 2017 showed that the majority of the public in Nigeria did not know the appropriate emergency numbers to call, and that trust in EMS systems is low [25]. The Lagos State Government website itself lists six different telephone numbers for LASAMBUS [26]. Furthermore, the existence of other emergency response systems, such as LASEMA or the LRU, can confuse both victims of trauma and innocent bystanders who are trying to help. Both of these EMS services also have multiple associated numbers and are listed ahead of LASAMBUS in the list of emergency telephone numbers on the Lagos government website [26]. The uncertainty of whom to call can result in LASAMBUS not being able to address the RTA or being delayed in its response. There has been an attempt to standardize and streamline the process with the introduction of an emergency communication network and call center [25]. However, multiple toll-free numbers (112, 123, etc.) continue to be advertised, impeding these efforts.

Our median response time from when LASAMBUS received the call to when they arrived on the scene (17 min) was comparable to the response time of ambulances in Accra, Ghana and is only 2 min longer than the median urban response time across all African EMS systems [15, 27]. Lower response times have been shown to be associated with better patient outcomes and higher chances of survival and is a crucial part of pre-hospital care management [27]. Findings from a study conducted in Spain in 2010 estimated that a 10 min reduction in response time could result in a 33% decrease in mortality rates in RTAs [28]. While further analysis into response time did not yield any significant associations, it is encouraging to note that our response time is similar to other LMICs in Africa. However, LASAMBUS has a self-identified goal of 10 min, which is 7 min faster than the current median response time, and highlights room for improvement.

The use of sirens by non-EMS vehicles is a challenge that LASAMBUS encounters and one that could add to this disparity. The inappropriate use of sirens, by governmental or military vehicles can desensitize the public, making them more likely to ignore LASAMBUS or other EMS vehicle sirens. Specific rules prohibiting the use of sirens by those other than emergency professionals can help to alleviate this. Also, the number of ambulance stations has increased from 18 in 2006 to 25 currently. Continuing to increase the number of ambulance stations will also decrease response time by increasing the proximity to RTA sites.

In relation to the third finding, we observed that a substantial proportion of the causes for delay reported by LASAMBUS was concerned with the public infrastructure, namely traffic congestion or poor access to RTA (65%), both of which were also significantly associated with specific outcomes. One explanation for these causes for delay could be the poor state of roads in Lagos. Narrow roads, potholes, or inadequate street lighting are all recorded problems in Lagos and can increase response time and traffic congestion, and obstruct access to the RTA [29]. In 2017, the Governor of Lagos outlined road construction and maintenance as a key priority in the annual budget [30]. The increasing prevalence of vehicle breakdowns and the influx of commercial buses and trucks amplify traffic congestion and its associated consequences as well [31]. In 2018, the Lagos State Traffic Management Agency (LASTMA) found that vehicular breakdowns in Lagos accounted for 70% of the traffic gridlocks in the state [31]. They theorized that strengthening the relationship between the public and LASTMA officials could more efficiently and effectively resolve these breakdowns [31].

One limitation of this study was incomplete forms, in which some fields were left blank. Examples of these fields include age and LGA. Despite this, we were able to successfully identify an outcome for all 1352 forms. In the future, we hope to link intervention forms to the ambulance points from which they were created and map the RTAs across LGAs. This will help to provide a more accurate picture of the distribution of RTA outcomes across Lagos. We are also in the process of exploring the causes for delay and response times in each LGA to detail specific points of intervention by LGA and ambulance point. Additionally, our future efforts will focus on piloting electronic data collection in high call-volume ambulances. We hope that this will improve the quality of data collection and standardize the intake process of LASAMBUS, to better track and improve victim outcomes.

## Conclusion

While the RTA mortality rate in Nigeria is increasing annually, Lagos is especially affected as one of the most populous states in Nigeria. LASAMBUS faces various obstacles in attending RTAs and its current response rate is alarming, playing a part in the increasing mortality rates. Focusing attention on reducing the occurrence of false calls, improving road conditions, and standardizing the contact methods for LASAMBUS will help to make this better. To achieve this, a collective effort has to be made by LASAMBUS, the Lagos Ministry of Health, and the Lagos Government. Future research on RTA patterns and victim outcomes by LGA will also help to further understand the circumstances influencing RTA mortality rates.

## Electronic supplementary material

Below is the link to the electronic supplementary material.

Supplementary file 1 (DOCX 28926 kb)
