# Supplementary figures and images for: Lagos state ambulance service: a performance evaluation
Source: Eur J Trauma Emerg Surg. 2020 Mar 10;47(5):1591–8. doi: 10.1007/s00068-020-01319-y (PMC8476380; doi:10.1007/s00068-020-01319-y)

Appendix A: Blank LASAMBUS Intervention Form


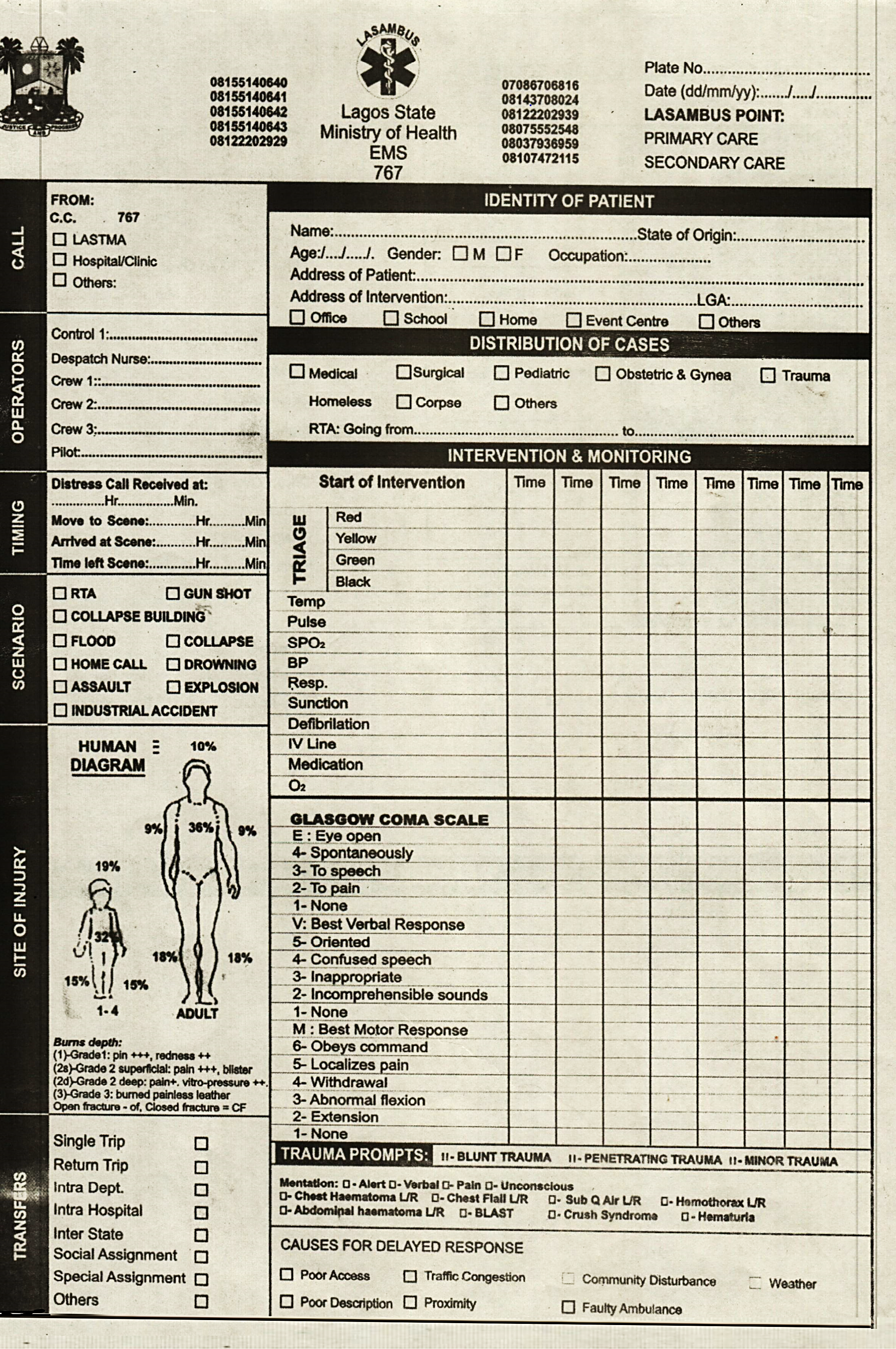


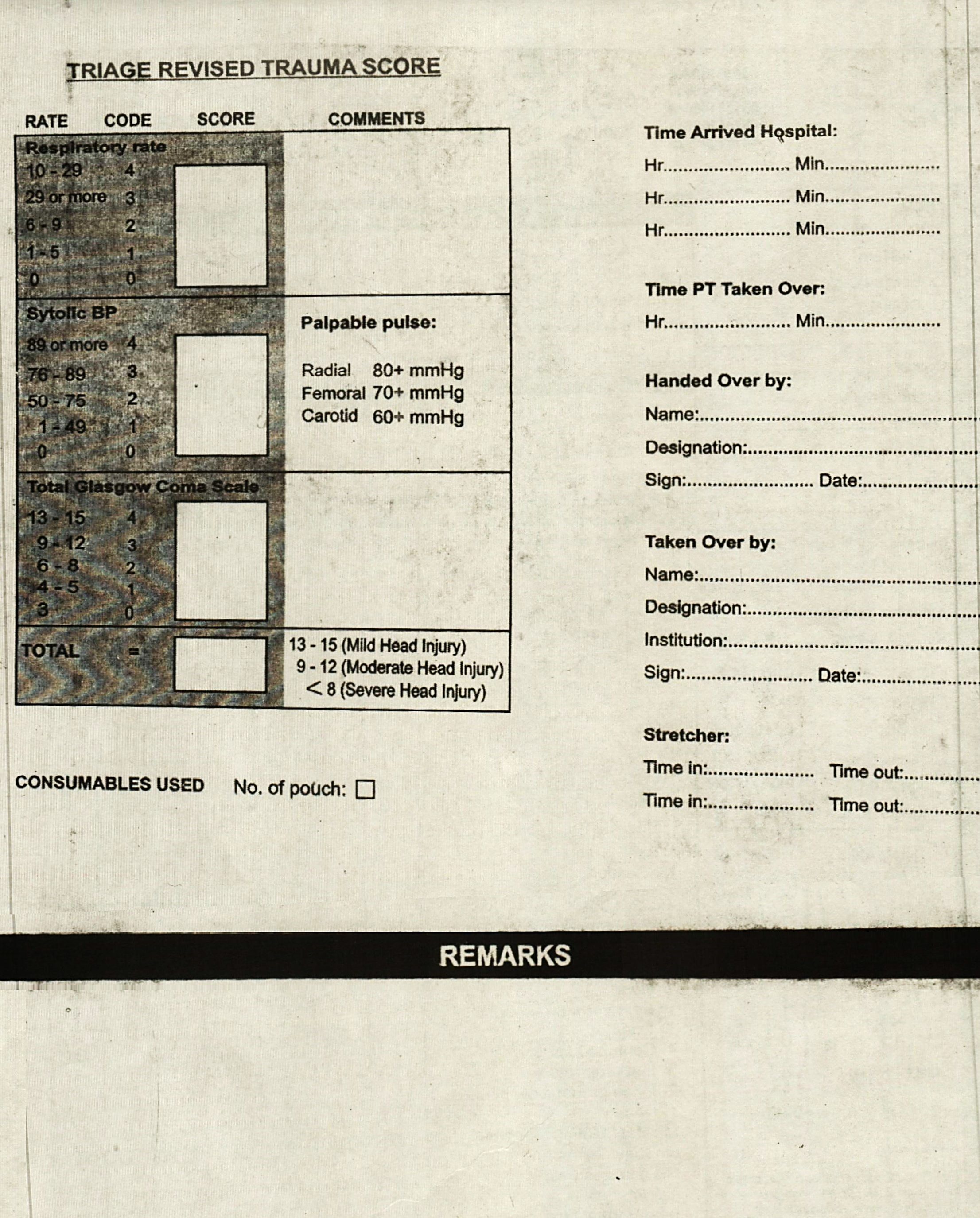

Supplement: Supplementary file 1 — (DOCX 28926 kb) [file 68_2020_1319_MOESM1_ESM.docx]
